# ANA‐negative severe lupus‐like presentation: Is it lupus or not?

**DOI:** 10.1002/ccr3.8145

**Published:** 2023-11-12

**Authors:** Shuangxi Li, Qi Bian

**Affiliations:** ^1^ Department of Nephrology Changhai Hospital, The Navy Medical University Shanghai China

**Keywords:** antinuclear antibody negative (ANA‐negative), epilepsy, renal failure, systemic lupus erythematosus (SLE)

## Abstract

**Key Clinical Message:**

Systemic lupus erythematosus is difficult to diagnose in patients who are antinuclear antibody (ANA) negative and lack typical clinical manifestations. For such patient who presented ANA‐negative severe lupus‐like manifestations, the diagnosis and treatment are a huge challenge. Histological findings may provide clues to diagnosis.

**Abstract:**

Systemic lupus erythematosus (SLE) is a multisystem autoimmune disease characterized by formation of autoantibodies to nuclear and cytoplasmic antigens. It was reported that a small subset of patients had typical clinical features of SLE with consistently negative antinuclear antibody (ANA), but such disease is usually mild and rarely involves multisystem. At present, there are no reports about severe lupus with ANA continued negative. Our report describes a 34‐year‐old Chinese woman who presented renal failure, multiple serous cavity effusion, and epilepsy, without malar rash, photosensitivity, lymphopenia, and arthritis. Further renal biopsy pathology revealed lupus‐like nephritis. Autoantibodies, including ANA, antibodies against Smith and against double stranded DNA, were negative. Such a ANA negative and lack of typical clinical symptoms of SLE patient, but with severe lupus‐like manifestations, whether it was lupus or not is worth discussing.

## Graphical abstract

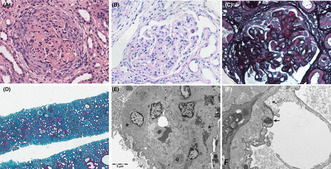

## 1 INTRODUCTION

Systemic lupus erythematosus (SLE) is a protean disease. The classification criteria have been evolving to increase the sensitivity and specificity of SLE diagnosis. The American College of Rheumatology (ACR) criteria (1982 version^1^ and 1997 revised version)^2^ have high specificity, but limited sensitivity. The Systemic Lupus International Collaborating Clinics (SLICC) 2012 criteria have increased sensitivity, but decreased specificity.^3^ The 2019 EULAR/ACR SLE classification criteria have both high sensitivity and specificity, and advocated positive ANA (ever) as an entry criterion.^4^, ^5^

The decision that ANA was used as a screening test and an entry criterion for SLE was made after the baseline facts had been worked up thoroughly. Nicolai Leuchten et al. showed ANA at a titer of 1:80 have 98% sufficiently high sensitivity in a systematic literature review and meta‐regression which included more than 13,000 SLE patients.^6^ A report showed that only 6.2% of patients were ANA negative among more than 1000 SLE patients who fulfilled the ACR classification criteria.^7^ Therefore, ANA is a useful and sensitive indicator for screening SLE. However, there are also a minority SLE patient with ANA negative.^8^, ^9^ It has been reported that among patients with “full‐house” or “Lupus‐like” nephropathy but negative serology for lupus, some of them developed autoantibodies and other clinical manifestations of SLE during the follow‐up, while some of them remained seronegative and developed no clinical findings of SLE other than full‐house nephropathy.^8^, ^10^ Another report describes three processes of autoantibodies in the development of lupus^11^: Stage 1: Patients had neither symptoms nor any detectable autoantibody levels; Stage 2: Patients develop detectable autoantibodies without clinical manifestations; Stage 3: Patients presented obvious clinical symptoms of lupus with autoantibodies positive.^11^ According to the above description, ANA is not only a diagnostic indicator of lupus, but also related to the progression of the disease.

Our report describes a young woman who presented an ANA negative and lack of typical clinical symptoms of SLE, but with severe lupus‐like manifestations. This is different from the previous reports about ANA‐negative lupus. At present, this is unique case report about severe lupus with ANA continued negative.

## 2 CASE REPORT

On December 8, 2020, a 34‐year‐old Chinese woman was found to have renal failure (creatinine of 778.4 μmol/L) due to frequent vomiting. She had not any medical history. On admission, her vital signs were as follows: blood pressure 190/100 mmHg, heart rate 108 bpm, respiratory rate 18 rpm, temperature 36.3°C, weight 50 kg, height 163 cm, and body mass index 18.8 kg/m^2^. On physical examination, she had conjunctival pallor and moderate edema of bilateral lower extremities. A few moist rales had been audible over both lung bases. The abdomen had been soft without tenderness. There were no other sighs including fever, hair fall, oral ulcers, and arthrodynia. Their nerve signs were normal. She had two healthy children.

**FIGURE 1:**
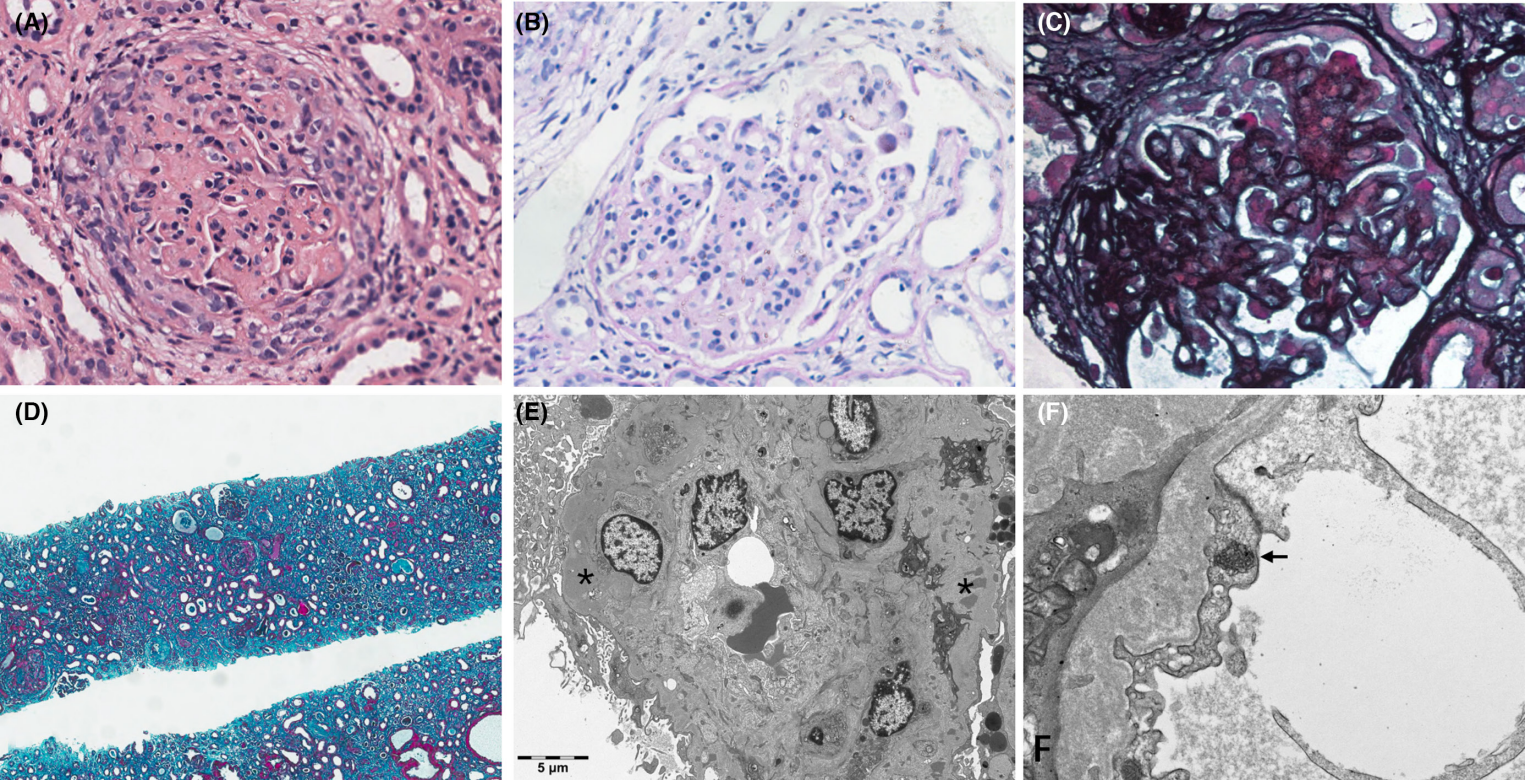
Renal pathological findings of the patient.

**FIGURE 2:**
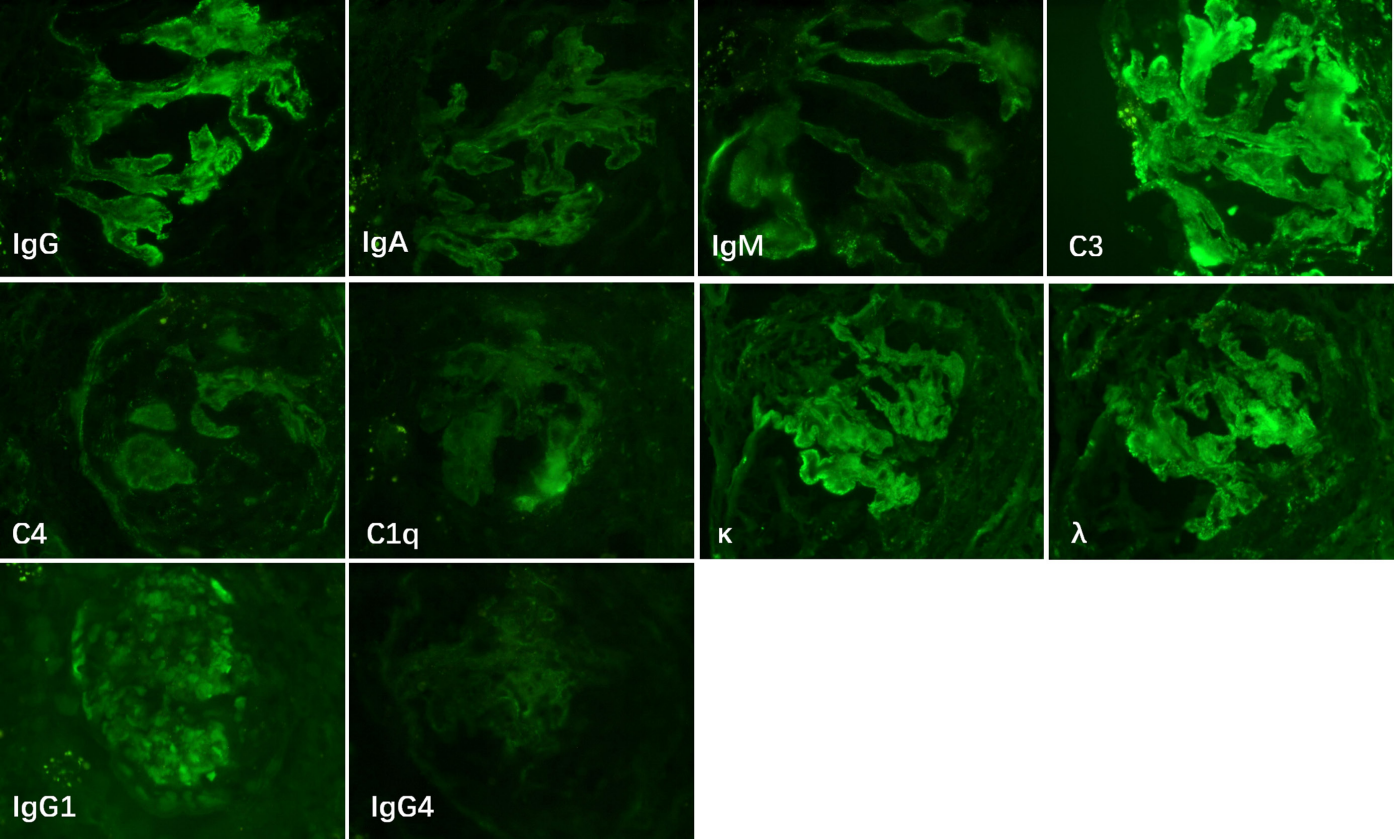
Immunofluorescence analysis of renal biopsy.

Initial investigation showed blood urea nitrogen 24.5 mmol/L (2.6–7.5), creatinine 808 μmol/L (41–73), eGFR (MDRD formula) 5.2 mL/min·1.73 m^2^, albumin 37 g/L (40–55), hemoglobin 88 g/L, erythrocyte sedimentation rate 88 mm/h (0–20), C‐reactive protein 0.4 mg/dL (0–0.3), calcium 2.10 mmol/L (2.11–2.52), phosphorus 2.62 mmol/L (0.85–1.51), parathyroid hormone 272 pg/mL, complement 3: 0.79 g/L (0.79–1.52), and complement 4: 0.41 g/L (0.16–0.38) (Table S1). Autoimmune antibodies test: antinuclear antibody (ANA), perinuclear antineutrophil cytoplasmic antibodies, myeloperoxidase antibodies, cytoplasmic antinuclear cytoplasmic antibodies, proteinase 3 antibodies, anti‐Sjogren antibody SSA and SSB, Smith (Sm) antibodies, glomerular basement membrane antibodies, double string DNA antibodies, and single string DNA antibodies: negative (Table S1). Cryoglobulins quantitative analysis: negative. Urinalysis showed proteinuria and hematuria. 24‐h urine protein level was 4259.2 mg (24 h urine volume: 1100 mL) (Table S1). Viral hepatitis profile and human immunodeficiency virus (HIV) were all negative. Echocardiography indicated pericardial effusion. A kidney biopsy was performed to confirm the etiology of the renal failure, which presented crescent glomerulonephritis, and multiple immune complexes deposition in the mesangial, subcutaneous, and subepithelial areas with membranoproliferative and “full‐house nephropathy” pattern (Figures 1 and 2).

**FIGURE 3:**
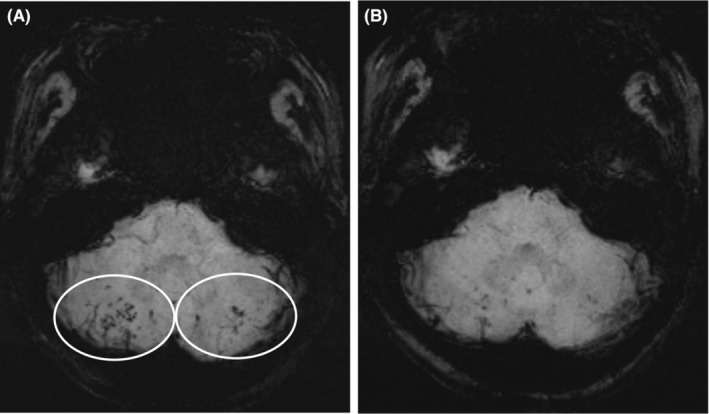
Susceptibility‐weighted imaging changes before and after treatment.

She was initiated on peritoneal dialysis due to renal failure. After kidney biopsy, she received intravenous methylprednisolone 80 mg, daily for 3 days. Thereafter, oral prednisone started at 40 mg per day (0.8 mg/kg/d) and intravenous cyclophosphamide 0.4 g (7.5 mg/kg) monthly. On January 18, 2021, she had a sudden, unprovoked seizure with loss of consciousness that lasted about 6 min. She had no previous history of seizures. Cerebrospinal fluid examination was normal. Magnetic resonance imaging of the brain revealed multiple abnormal signals in cerebral cortex, basal ganglia, and cerebellar hemispheres. Magnetic resonance angiography was negative. Susceptibility‐weighted imaging showed hemosiderosis deposits in cerebellar hemisphere and occipital lobe (Figure 3). Those results showed that the seizures maybe related to cerebral vasculitis. Autoantibody reexamination was still negative, but C3 (0.43 g/L) and C4 (0.11 g/L) were decreased significantly (Table S1). The patient received Debarkin and pulse intravenous injection of immunoglobulin 20 g (0.4 g/kg) daily for 5 days. She then received pulse methylprednisolone 500 mg for 3 days and turned to oral prednisone 40 mg/day, and hydroxychloroquine (HCQ) was also chosen to treatment. But given that the subsequent serious pulmonary bacterial infection, fungal enteritis, and severe myelosuppression, she was no longer treated with cyclophosphamide. After infection control, HCQ and low‐dose mycophenolate mofetil was chosen as maintenance treatment. The prednisolone taper was continued to a dose of 5 mg/day. The treatment is effective, and the brain lesion was obviously shrinked after 6 months (Figure 3). She had no further seizures and no other specific clinical symptoms within more than 3 years of clinical follow‐up. CARE guidelines and methodology have been followed in this study.

## 3 DISCUSSION

The patient presented with chronic renal failure at the time of diagnosis, and there was a lack of evidence and clinical manifestations of secondary renal failure. If the patient was directly treated with renal replacement therapy without renal biopsy, it would be more difficult to diagnose. For this patient, renal pathology is an important basis for lupus diagnosis. SLICC used full‐house staining as the sole criteria to diagnose SLE.^3^ However, it has been reported that the prevalence of no‐lupus full‐house nephropathy was 20%–30%.^12^, ^13^, ^14^, ^15^ Pathologies of full‐house nephropathy should also be considered in the differential diagnosis of SLE, including primary glomerular diseases (membranous nephropathy, C1q nephropathy, IgA nephropathy), infections (endocarditis, HIV, HBV, HCV, BK, and CMV virus), diabetes mellitus, and liver diseases.^12^, ^13^, ^14^ This patient had none any evidence of these disease. Another report showed that five renal pathological features for enhanced the sensitivity and specificity of stand‐alone kidney biopsy to diagnose lupus nephritis, which included “full‐house” staining, intense C1q staining, extraglomerular deposits, combined subendothelial and subepithelial deposits, and tubuloreticular inclusions, were selected to create a scoring system to define lupus nephritis with reasonably high sensitivity and specificity.^16^ According to this scoring system, this patient had 3 scores: “full‐house” staining, subendothelial and subepithelial deposits, and tubuloreticular inclusions, with sensitivity of 80% and specificity of 95% for the diagnosis of lupus nephritis.

However, there is still a certain bias in the diagnosis of LN by renal biopsy pathology alone. In the SLICC classification, if patients are ANA negative, however, they have to fulfill at least four of the other 10 (or 16) criteria, also biasing against ANA‐negative SLE.^3^ At the onset, the patient presented with multiple serous effusion, renal failure, which had not yet met the diagnosis of ANA‐negative SLE. During the follow‐up, the patient developed new signs: epilepsy and hypocomplement. Given excluding other possible diseases, combined with the patient's four clinical symptoms and lupus‐like renal pathological findings, the diagnosis of ANA‐negative SLE is considered, and the treatment options taken for SLE are effective and the extra‐renal symptoms are controlled, although unfortunately the long‐term renal replacement therapy is needed.

Therefore, for patients lacking typical clinical symptoms, the auxiliary role of pathological manifestations in some intractable cases is crucial. ANA screening for SLE is a good tool, but autoantibody testing should not be overly relied upon when both pathological and clinical findings support the diagnosis of SLE.

## 4 CONCLUSION

This patient is a unique case of ANA‐negative severe lupus. For such cases, it is easy to be missed or misdiagnosed, which is a challenge in diagnosis and treatment. How to detect these diseases early still needs more research. According to our case report, kidney biopsy needs to be appreciated especially in patients with unexplained renal failure.

## AUTHOR CONTRIBUTIONS

**Shuangxi Li:** Writing – original draft; writing – review and editing. **Qi Bian:** Resources; supervision.

## FUNDING INFORMATION

None.

## CONFLICT OF INTEREST STATEMENT

The authors have no conflicts of interest.

## CONSENT

A written consent was obtained from the patient for the publication of the case.

## Supporting information


Table S1:

## Data Availability

We agree to make the manuscript available to general people and are also ready to provide other necessary data regarding the manuscript in case required.
