# Supplementary material for: ANA‐negative severe lupus‐like presentation: Is it lupus or not?
Source: Clin Case Rep. 2023 Nov 12;11(11):e8145. doi: 10.1002/ccr3.8145 (PMC10641295; doi:10.1002/ccr3.8145)
Supplement: Supplementary file 1 — Table S1: [file CCR3-11-e8145-s001.docx]

|  | | normal reference range | Dec 8th 2020 | Jan 18th 2021 | Mar 12th 2021 | Oct 9th 2021 |
| --- | --- | --- | --- | --- | --- | --- |
| **Renal function test** | Creatinine  (mmol/L) | 41-73 | 808 | 466 | 646 | 751 |
|  | eGFR (MDRD formula) ml/min·1.73m2 | 80-120 | 5.2 | 9.8 | 6.8 | 5.7 |
|  | BUN(mmol/L) | 2.5-5.7 | 24.5 | 20.1 | 19.6 | 21.8 |
| **blood routine examination** | Hb (g/l) | 130-175 | 88 | 69 | 91 | 120 |
|  | WBC (×10^9^) | 3.5-9.5 | 4.67 | 5.14 | 4.18 | 8.51 |
|  | PLT (×10^9^) | 125-350 | 297 | 126 | 112 | 158 |
| (ESR) (mm/h) | | ＜15 | 88 | - | - | - |
| Calcium (mmol/L) | | 2.11-2.52 | 2.10 | 1.81 | 2.04 | 2.36 |
| phosphorus (mmol/L) | | 0.85-1.51 | 2.62 | 2.51 | 1.32 | 0.95 |
| PTH (pg/ml) | |  | 272 |  | 7.82 | 72.5 |
| Urinary protein (mg/24h) | | 28-141 | 4259.2 | - | - | - |
| **complement** | C4 (g/L) | 0.16-0.38 | 0.41 | 0.11 | 0.12 | 0.33 |
|  | C3 (g/L) | 0.79-1.52 | 0.79 | 0.43 | 0.32 | 0.67 |
| **Autoimmune antibodies test** | ANA | negative | negative | negative | negative | negative |
|  | p-ANCA | negative | negative | negative | negative | negative |
|  | MPO（RU/ml） | ＜20 | ＜20 | ＜20 | ＜20 | ＜20 |
|  | c-ANCA | negative | negative | negative | negative | negative |
|  | PR3（RU/ml） | ＜20 | ＜20 | ＜20 | ＜20 | ＜20 |
|  | SSA and SSB | negative | negative | negative | negative | negative |
|  | Smith (Sm) antibodies | negative | negative | negative | negative | negative |
|  | GBM（RU/ml） | ＜20 | ＜20 | ＜20 | ＜20 | ＜20 |
|  | ds DNA（IU/ml） | ＜100 | 20.3 | 18.7 | 22.6 | 23.8 |
|  | ss DNA（RU/ml） | ＜20 | 12.9 | 6.8 | 12 | 11.9 |
| Cryoglobulins quantitative analysis | | negative | negative | - | - | - |
| **Infection Indicators** | Hepatitis B and C | negative | negative | - | - | - |
|  | HIV | negative | negative | - | - | - |

**supplementary table 1: main clinical laboratory test result**

BUN: blood urea nitrogen; Hb: Hemoglobin: WBC: white blood cell; PLT: platelet; PTH: parathyroid hormone;

ESR: erythrocyte sedimentation rate; ANA: antinuclear antibody; p-ANCA: perinuclear antineutrophil cytoplasmic antibodies; MPO: myeloperoxidase; c-ANCA: cytoplasmic antinuclear cytoplasmic antibodies; PR3: proteinase 3; GBM: glomerular basement membrane; ds DNA: double string DNA; ss DNA: single string DNA; SSA: Sjogren syndrome A; SSB: Sjogren syndrome B; HIV: human immunodeficiency virus.
